# Risk and protective factors associated with health risk behaviours among school learners in Western Cape, South Africa

**DOI:** 10.1186/s12889-022-14845-8

**Published:** 2023-01-04

**Authors:** Godswill N. Osuafor, Chinwe E. Okoli, Reamogetse Phateng

**Affiliations:** 1 Department of Population Studies and Demography, North West University, Mafikeng Campus, Mahikeng, South Africa; 2 Federal Medical Centre, Umuahia, Nigeria

**Keywords:** High school, Alcohol use, Cannabis, Sexual intercourse, Longitudinal, Health-risk behaviour

## Abstract

**Background:**

Health risk behaviour is rife among school learners in the Western Cape province. This paper assesses risk and protective factors related to health risk behaviours among high school learners. Method: Longitudinal data were sourced from 2950, 2675 and 2230 at Time 0, Time 1 and Time 2 among grade 8 learners aged 13–18 years between 2012 and 2013. Health risk behaviours were assessed on alcohol consumption, smoking cannabis in the past six months, and ever having sexual intercourse. The sociodemographic variables examined were age, sex, residence, socioeconomic status (SES), family structure and population group. Contextual variables studied were the feeling of learners about the intervention program, participation in religious activities, paid casual work and school sports. Descriptive statistics, bivariate associations and binary logistic analyses predicting health risk behaviours were carried out using generalized linear mixed models after restructuring the data collected at different time points.

**Result:**

Health risk behaviours increased consistently for alcohol consumption (25.7–42.7%), smoking cannabis (10.4–22.1%) and (22.3–36.0%) engaging in sexual intercourse. Increasing age emerged as a risk factor for all the health risk behaviours: alcohol consumption [OR:1.3 (1.2–1.4), *p* < 0.001]; smoking cannabis [OR:1.3 (1.2–1.4), *p* < 0.001] and had sex [OR:1.5 (1.4–1.7), *p* < 0.001]. Participation in paid casual work also predicted health risk behaviour: alcohol use [OR:1.5 (1.2–1.8), *p* < 0.001]; smoking cannabis [OR:1.3 (1.0-1.7), *p* < 0.05] and sex [OR:1.4 (1.1–1.7), *p* < 0.01]. High SES and feelings about the EPEP programme enhanced alcohol consumption and smoking cannabis. Smoking cannabis was augmented by residing in an urban area. Participation in school sports was associated with increased alcohol consumption and engaging in sexual intercourse. Participation in religious activities was protected against alcohol consumption [OR:0.7 (0.53–0.83), *p* < 0.001]; and sex [OR: 0.5 (0.4–0.7), *p* < 0.001]. Being a female and belonging to a coloured population group diminished engaging in sexual intercourse, and the family structure of both parents attenuated involvement in smoking cannabis.

**Conclusion:**

The findings of the study on risks and protective factors on health risk behaviours mirror those of school-based programmes in developing countries. Learners who participated in paid work and school sports are at risk of adverse health outcomes. Furthermore, participation in religious practices and family structure roles in attenuating health risk behaviours should be integrated and considered in the school-based intervention programme.

## **Introduction**

Indulging in alcohol, substance use, and early sexual initiation are major health risks for adolescents worldwide. Globally, over a quarter (26.5%) of all 15–19-year-old are current drinkers, amounting to 155 million adolescents [1, 2]. Evidence from school-based surveys on country-level data showed that one in four 13 to 15-year old used alcohol during the last 12 months; one in every ten girls and one in every five boys used tobacco, with lower rates for cannabis [3]. In 2017, a systematic review and meta-analysis on substance use revealed that 25.7% of adolescents used cannabis in Southern Africa [4]. National surveys revealed the use of cannabis ranged from 2 to 9% among adolescents [5]. National Youth Risk Behaviour Survey (YRBS), revealed that binge drinking increased among adolescents between 2002 and 2008 [6]. School-based surveys have shown rates of 22% and 39.9% on ever-consumed alcohol in cross-sectional [7] and longitudinal [8] studies respectively in South Africa. South African National HIV Prevalence, Incidence and Behaviour Survey have shown an increase in early sexual debut before the age of 15 between 2008 and 2017 [9]. A risk-reduction intervention pilot study among adolescent females showed that at baseline, 94% of participants tested positive for cannabis, 17% were HIV-positive and 11% were pregnant in Cape Town [10].

Risk behaviours among adolescents are a gateway to a host of negative health outcomes such as sudden death [11, 12], contraction of sexually transmitted diseases including HIV [10], unplanned pregnancy [10, 13] and vulnerability to injury [8]. For instance, 170.9 million, 85.0 million, and 27.8 million disability-adjusted life-years (DALYs) were consequences of tobacco, alcohol and illicit drug use respectively in 2017 [14]. In addition, mortality rates of 110.7, 33.0, and 6.9 deaths per 100,000 people were attributed to tobacco, alcohol, and illicit drugs, respectively [14].

Studies have shown that older adolescents had symptoms of sexually transmitted infections (STIs) compared with younger counterparts [15]. Other studies revealed that females though vulnerable to coerced sex were less concerned about contracting HIV [16]. In 2018, about 33,000 girls compared to 4,200 boys aged 10–19 years were HIV-positive in South Africa [17]. These differentials in HIV prevalence between female and male adolescents may be attributed to the attitude of adolescent girls to HIV infection in South Africa. High rates of sexually transmitted infections among young people were not restricted to South Africa but have been reported in pacific regions [18].

Several studies have demonstrated that sociodemographic variables (male and increasing age of adolescents) [16, 19–23] were risk factors associated with involving in early sexual debut and sexual activity. Other risk factors for sexual behaviour among adolescents were low socioeconomic status (SES) [24], and substance use [19, 23, 25, 26]. Some studies failed to show a clear pattern of associations between SES and alcohol consumption or marijuana use [27, 28]. In general, adolescent risk behaviours have been associated with parent risk behaviour [20, 29], Peer pressure [20, 30–33], geo locality [34–36], availability of alcohol [37, 38], socioeconomic privilege [39] and substance use [21, 23].

Conversely, factors that enhanced negative behaviours exerted protective effects in other studies. For instance, studies have revealed that parental/guardian and positive peer support [19, 40] played protective roles against adolescents’ risk of sexual activity. Parental/guardian and positive peer support also reduced the tendency to smoke cigarette smoking among school-going adolescents [26]. In addition, parental support showed protective effects against illicit drug use in South Africa [41] and Morocco [42]. Furthermore, studies have demonstrated that religious beliefs strongly reduce the likelihood of alcohol consumption [35, 43] and substance use [35]. Other studies have demonstrated that religiosity [44] and family connectedness [19, 44] lowered adolescents’ tendency to engage in sexual risk behaviour.

Evidence of risk and protective health factors on adolescents’ health risk behaviours has produced mixed results. Substances use and early sexual initiation have debilitating consequences. Ensuring a safe school environment requires empirically established risk and protective factors influencing adolescents’ disposition to risky behaviours. School-based interventions to assuage risk and enhance protective factors against health risk behaviours have been initiated in South Africa. Given the severity of alcohol consumption, illicit substance use and early sexual activities among high school learners, school-based health intervention programmes need to be examined to deduce risks and protective factors to address health risk behaviours. Therefore, the study aimed to identify risk and protective factors against health risk behaviours among high school learners in Western Cape.

## **Methods**

### The study method

The evaluation of peer education programme (EPEP) was a longitudinal study rolled out in 236 schools to measure the change in attitudes, knowledge and behaviour among Grade 8 learners in Western Cape schools. The schools where the EPEP curriculum was implemented formed the intervention group or otherwise the control group. Data were collected from 2950, 2675 and 2230 learners of the age range 13 to 18 at three-point times. The EPEP was carried out between February 2012 and June 2013. The evaluation was done at the end of the intervention programme. Baseline data were collected at the time (Time 0) and subsequently, from the same learners, the intervention programme was initiated in the schools. Immediately after the intervention elapsed, data were collected at the time (Time 1). After five to seven months post-intervention, data were collected at the time (Time 2). The EPEP curriculum called ‘Listen Up’ has seven structured lessons designed to run concurrently with life orientation lessons for 35 to 45 min. The main content of the curriculum was homies and helpers; making smart decisions; For the love of it: relationships; Safe my mate: reducing risk; no go waiting for sex; teenage pregnancy and how much is too much: talking about drinking. Data on health risk indicators about peer education, substance use, HIV/AIDS and sociodemographic variables were gathered. A self-administered survey questionnaire that was translated and back-translated and piloted prepared in English, Afrikaans and Xhosa was used for data collection.

Respondents who participated in the peer education programme were coded 1 while the control group was coded 0. Learners were asked “how do you feel about the Peer Education Programme? This was rated on a scale of on a scale (1 = very disappointed, 2 = disappointed, 3 = I don’t know, 4 = excited 5 = very excited). Responses 4 and 5 were merged and coded, 1 = excited and 2 were merged and coded 2 = disappointed while response 3 was coded 3 = Don’t know. Information on the sociodemographic characteristics of the learners was sourced on age, sex, place of residence, population group, family structure and socioeconomic status (SES). Age was treated as a continuous variable. Discrete variables were categorized: Sex (1 = male, 2 = female); Place of residence (1 = rural, 2 = urban); population groups (1 = black, 2 = coloured and 3 = white) and family structure (1 = single parents, 2 = both parents and 3 = grandparents).The SES variables were measured from the responses on basic human needs: ^1^. We don’t have enough money for food; ^2^. We have enough money for food, but not other basic items such as clothes; ^3^. We have enough money for food and clothes but are short for other things and ^4^. We have enough money for food and clothes, and also a bit extra for other things. Responses 1 and 2 were categorized as poor SES; responses 3 and 4 were categorized as middle and high SES respectively. Individual factors were measured on participation in religious activities, casual work and school sports. Three questions were posed to the learners. Learners were asked, “how many days a week do you go to church or choir or youth group or Muslim school?” How many days a week do you do casual work for which you get paid? How many days a week do you participate in sports - at school? Each of these questions was measured on a scale of 1 to 3: 1 = never; 2 = a few times a week and 3 = every day of the week. Response 1 was coded 0 which means “never” while 2 and 3 were merged and coded 1 as “always”.

### Health risk behaviour

Three dimensions of health risk behaviours were assessed: alcohol consumption, smoking of cannabis and sexual intercourse. Respondents were asked whether they have ever consumed alcohol in the past six months; ever consumed cannabis in the past six months and if they have ever had sex. The responses were coded 0 if the learner never consumed alcohol or smoke cannabis in the past six months. If the learner responded “yes” to alcohol consumption or smoking of cannabis, it was coded 1. Learners reporting never had sex were coded 0 whereas “yes” was coded 1.

### Data analysis

All statistical analyses were performed using IBM Statistical Package for Social Sciences (SPSS) version 26. Data were restructured and all analyses were executed using generalized linear mixed models. Descriptive statistics were used to process the health risk behaviour and sociodemographic characteristics. Bivariate associations between health risk behaviours and sociodemographic characteristics were assessed using the Chi-Square test. Binary logistic regression analyses were carried out to detect risk and protective factors associated with alcohol consumption, smoking cannabis and sexual intercourse. The findings were presented as odds ratio and confidence interval. In all the tests, *p*-values of < 0.05 were considered statistically significant.

## **Results**

### Descriptive statistics

Table 1 below showed that the mean age of the respondents in the peer education evaluation was 14.03 years (SD = 1.2). About 79.0% who participated in EPEP were in the intervention group. On the views of the learners about the EPEP programme, 43.1% were excited and one in 10 were disappointed whereas 46.3% were uncertain of their feelings about the programme. Over half (56.6%) were females and the majority (80.3%) of the respondents were living in the urban area. Slightly over half (53.0%) of the respondents were black population. Socioeconomic status showed that about a quarter (24.4%) and 45.5% belong to low SES and high SES respectively. In terms of family structure for caregiving, half (50.9%) were living with a single parent, 30.5% were receiving care from two parents and less than a quarter were supported by grandparents and others. The majority (78.1%) of the respondents engage in religious activities regularly, about 43.3% of the learners engage in casual work with remuneration and about two-thirds participated in school sports on regular basis.

**Table 1:** Distribution of learners by background characteristics

	**Number of observations (N)**	**Percent (%)**
**Age**		
< = 13	2397	34.51
14	2398	34.53
15 +	2150	31.96
**EPEP Programme**		
Control	1683	20.97
Intervention	6342	79.03
**Feeling about the EPEP Programme**		
Excited	2744	43.05
Disappointed	679	10.65
Don't Know	2950	46.30
**Sex**		
Male	3786	43.44
Female	4929	56.56
**Place of residence**		
Rural	1450	19.75
Urban	5895	80.25
**Population group**		
Black	4416	53.02
Coloured	3573	42.91
White	339	4.07
**Socioeconomic status**		
Low	1792	24.43
Middle	2207	30.10
High	3335	45.47
**Family structure -Caregiver**		
Single parents	3365	50.87
Both parents	2018	30.52
Grandparents	1231	18.61
**Go to church or choir or youth group or Muslim school**		
Never	1583	21.91
Always	5643	78.09
**Do casual work for which you get paid**		
Never	4083	56.74
Always	3113	43.26
**Participate in sports—at school**		
Never	2716	38.45
Always	4346	61.55

### Health Risk behaviours

**Fig. 1:**
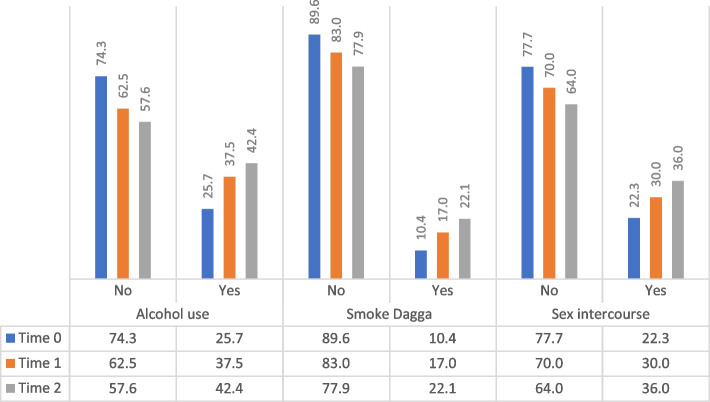
Percentage of health risk behaviour among the school learners in Western Cape

Figure 1 below showed the percentage distribution of health risk behaviour among the learners. All three measures of health risk behaviour showed a consistent increase from time 0 to time 2. In terms of alcohol consumption, the proportion of learners who consumed alcohol increased from 25.7 to 42.4% between the two intervals. Smoking Cannabis increased from 10.4 to 22.1%, which suggests a doubling between the two-time points. Similarly, the percentage of learners engaging in sexual intercourse increased from 22.3 to 36.0% between the two intervals.

### Bivariate analyses

Table 2 below showed the association of health risky behaviour with independent variables among learners. The association between alcohol consumption and background profile showed significant differences except for the EPEP intervention programme, family structure and participation in school sports. Alcohol consumption increased consistently with increasing age. Learners who stated that they were “excited” with the EPEP programme showed the lowest percentage of reported alcohol consumption. The proportion of male and urban dwellers who consumed alcohol was higher than female and rural peers. In terms of population group and SES, the coloured, and the high SES showed the highest proportion of reporting alcohol consumption. Compared to learners who engage in religious activities, those that never engaged in religious activities showed a higher percentage of reporting alcohol consumption. Furthermore, learners who did causal work with remuneration were more likely to consume alcohol than those who did not do causal work.

**Table 2:** Association of health risk behaviours with independent variables among learners

	**Alcohol use N (%)**	**Use cannabis N (%)**	**Had sex N (%)**
**Age**	***	***	***
< = 13	645 (27.6)	239 (10.2)	383 (17.0)
14	800 (34.1)	345 (14.8)	580 (25.9)
15 +	890 (42.4)	493 (24.0)	858 (43.8)
**EPEP Programme**			
Control	458 (35.5)	219 (17.1)	365 (30.0)
Intervention	1706 (35.1)	791 (16.3)	1338 (28.9)
**Feeling about the EPEP Programme**	***	***	***
Excited	792 (29.5)	377 (14.2)	709 (27.7)
Disappointed	234 (35.7)	130 (20.2)	232 (39.1)
Don't know	1031 (35.7)	495 (17.2)	791 (29.2)
**Sex**	***	***	***
Male	893 (35.4)	452 (18.0)	836 (35.7)
Female	1049 (31.0)	455 (13. 6)	728 (22.5)
**Place of residence**	***	**	**
Rural	404 (28.7)	179 (12.9)	421 (32.5)
Urban	2057 (35.7)	951 (16.6)	1546 (28.1)
**Population group**	**	*	***
Black	846 (31.1)	394 (14.7)	780 (30.8)
Coloured	930 (34.8)	438 (16.5)	633 (24.8)
White	79 (28.8)	32 (11.5)	72 (28.2)
**Socioeconomic status**	***	**	***
Low	519 (29.7)	250 (14.5)	545 (34.0)
Middle	658 (30.8)	309 (14.6)	629 (31.4)
High	1269 (38.8)	579 (17.8)	782 (24.8)
**Family structure -caregiver**		***	***
Single parents	1121 (34.2)	520 (16.0)	938 (30.2)
Both parents	643 (32.4)	259 (13.1)	471 (25.1)
Grandparents	436 (36.4)	252 (21.2)	339 (30.1)
**Go to church or choir or youth group or Muslim school**	***	***	***
Never	663 (42.9)	322 (21.0)	548 (37.7)
Always	1772 (32.1)	781 (14.2)	1347 (25.7)
**Do casual work for which you get paid**	***	***	***
Never	1224 (30.6)	542 (13.6)	975 (25.6)
Always	1204 (39.7)	552 (18.3)	909 (31.8)
**Participate in sports—at school**			***
Never	891 (33.4)	393 (14.8)	593 (23.3)
Always	1492 (35.2)	685 (16.2)	1258 (31.6)

^*^p < 0.05; **p < 0.01; ***p < 0.001

Apart from the EPEP intervention programme and participation in school sports, smoking cannabis showed a significant association with all the background characteristics (Table 2). Smoking cannabis increased consistently with age. Males and those learners living in urban areas were more likely to report smoking cannabis compared with females and rural counterparts. The proportion reporting smoking cannabis was highest (16.5%) and (17.8%) for the coloured and high SES respectively. Learners who were taken care of by both parents and those who indicated that the EPEP programme was exciting showed the lowest percentages in reporting smoking cannabis. Those learners who participated in religious activities were less likely to smoke cannabis compared with learners who never participated in religious activities. Conversely, learners who did casual work were more likely to report smoking cannabis than those who did not do any casual work.

Table 2 further showed that reporting sexual intercourse was associated with the background characteristics except for the EPEP intervention programme. The tendency to engage in sexual intercourse increases with advancement in age. Learners who deemed the EPEP programme to be exciting showed the lowest percentage of reporting that they engaged in sexual intercourse. Learners who were males and those living in urban areas were more likely to engage in sexual intercourse compared with females and those residing in rural areas. Proportion reporting that they engaged in sexual intercourse was lowest among the coloured population group, those from high SES socioeconomic strata and learners whose parents were their caregivers. Participating in religious activities was associated with a lower likelihood of engaging in sexual intercourse. Furthermore, learners who neither did casual work nor participated in school sports reported a lower tendency to engage in sexual intercourse than those who did casual work and participated in school sports.

### Multivariate analyses

The result of multivariate binary logistic regression analyses is shown in Table 3 below. Statistically significant risk factors for alcohol consumption were age, unsure about the feeling of the EPEP programme, living in an urban area, high SES, and participating in paid casual work and school sports. The only protective factor against alcohol consumption was regular participation in religious activities. The likelihood to consume alcohol was increased by 29% (*p* < 0 0.001) due to an increase in age. The odds of consuming alcohol increased significantly by 33% (*p* < 0.05) and 75% (*p* < 0 0.001) for urban and high SES learners compared with their rural and low SES peers respectively. Learners who stated that they did not know how they felt about the EPEP programme were 1.4 times more like to consume alcohol compared with those who stated that the EPEP programme was exciting. The odds of consuming alcohol for learners who did casual work with remuneration and those that participated in school sports were 1.5 times and 1.3 times higher than the expected odds for learners who neither did casual work nor participated in sports. The odds of consuming alcohol were significantly reduced by 33% (*p* < 0 0.001) for learners who participated in religious activities compared to those who never participate in religious practices. However, the EPEP intervention programme, sex, population group and caregiver did not show significant association as risk or protective predictors against alcohol consumption.

**Table 3:** Multivariate analyses of health risk behaviours and risk and protective predictors

	**Alcohol use**		**Smoke Cannabis**		**Had Sex**	
	**Odds ratio**	**95% CI**	**Odds ratio**	**95% CI**	**Odds ratio**	**95% CI**
**Age**	1.29***	1.19–1.40	1.29***	1.17–1.44	1.49***	1.36–1.65
**EPEP Programme**						
Control (ref)	1.00		1.00		1.00	
Intervention	0.98	0.77–1.24	1.19	0.86–1.63	1.15	0.88–1.51
**Feeling about the EPEP programme**						
Excited (ref)	1.00		1.00		1.00	
Disappointed	1.29	0.91–1.84	1.71**	1.14–2.56	1.39	0.95–2.04
Don't Know	1.40**	1.15–1.71	1.20	0.93–1.55	1.09	0.88–1.34
**Sex**						
Male (ref)	1.00		1.00		1.00	
Female	0.94	0.77–1.14	0.94	0.74–1.19	0.74**	0.60–0.91
**Place of residence**						
Rural (ref)	1.00		1.00		1.00	
Urban	1.33*	1.03–1.73	1.58*	1.11–2.24	1.04	0.79–1.36
**Population Group**						
Black (ref)	1.00		1.00		1.00	
Coloured	1.15	0.94–1.39	1.07	0.84–1.38	0.80*	0.65–0.99
White	1.01	0.65–1.59	0.94	0.52–1.70	0.96	0.59–1.55
**Socioeconomic Status**						
Low (ref)	1.00		1.00		1.00	
Middle	1.25	0.95–1.65	1.05	0.74–1.49	0.92	0.69–1.23
High	1.75***	1.34–2.28	1.55**	1.12–2.15	0.88	0.66–1.16
**Family structure -Caregiver**						
Single parents (ref)	1.00		1.00		1.00	
Both parents	0.85	0.683–1.06	0.75*	0.56–0.99	0.91	0.72–1.16
Grandparents	1.04	0.81–1.33	1.30	0.96–1.75	0.83	0.63–1.09
**Participate in religious Activities**						
Never (ref)	1.00		1.00		1.00	
Always	0.67***	0.53–0.83	0.76	0.57–1.00	0.52***	0.41–0.65
**Get paid for Casual work**						
Never (ref)	1.00		1.00		1.00	
Always	1.48***	1.23–1.79	1.32*	1.04–1.68	1.40**	1.14–1.73
**Participate in school sport**						
Never (ref)	1.00		1.00		1.00	
Always	1.27*	1.04–1.56	1.23	0.95–1.58	1.68***	1.36–1.65

^*^p < 0.05; **p < 0.01; ***p < 0 .001; *Ref*  reference category; *CI*  confidence interval

The risk factors associated with smoking cannabis were age, being disappointed with the EPEP programme, living in an urban area, high SES and getting paid for casual work. Caregiving by both parents was the strongest protective factor against smoking cannabis. Participation in religious activities was a protective predictor against smoking cannabis though weakly significant (OR = 0.76, *p* = 0.054). The odds of smoking cannabis increased by 29% (*p* < 0 0.001) for age. Reporting disappointment with the EPEP programme was 1.7 times higher for smoking cannabis than the expected odds for stating that the EPEP programme was exciting. The odds of smoking cannabis increased by 58% (*p* < 0.05) and 55% (*p* < 0.01) for urban and high SES learners compared with their rural and low SES peers respectively. The odds of smoking cannabis were reduced by 25% (*p* < 0.05) for learners whose both parents provided care compared with single parents. Learners who engaged in casual work with payment were 1.3 times more like than those who never worked for pay to smoke cannabis. However, EPEP intervention programme, sex, population group and participation in school sports did not emerge as significant risk or protective factors against smoking cannabis.

Engaging in sexual intercourse was significantly associated with age, getting paid from casual work, and participation in school sports as risk factors. Being a female, belonging to a coloured population group and participating in religious activities were protective against engaging in sexual intercourse. The odds of engaging in sexual intercourse increased significantly by 49% (*p* < 0 0.001) for an increase in age. Similarly, learners who did casual work and those who participated in school sports were 1.4 times and 1.68 times more likely to indulge in sexual intercourse than their peers who never worked or participated in sports. Conversely, the odds of engaging in sexual intercourse were reduced by 26% ( *p* < 0.01) and 20% (*p* < 0.05) for females and coloured compared with males and black respectively. Similarly, the odds of having sexual intercourse were reduced by 48% (*p* < 0 0.001) for learners who regularly participated in religious activities compared with those who never participated in religious practices. EPEP intervention programme, feeling about the EPEP programme, residence, SES, and the family structure did not show significant association with learners engaging in sexual intercourse.

## **Discussion**

The study aimed to assess the prevalence of health risk behaviours and identify risk and protective factors driving these behaviours among a cohort of grade 8 learners. The programme was to prevent health risk behaviours among the learners. However, the prevalence of alcohol use, smoking cannabis and engaging in sexual intercourse increased over time. The reasons were not clear from the dataset. However, we assume that an intervention programme may not overtly stop learners’ tendency to experiment or explore certain behaviour. These experimentations and explorative behaviours constitute a developmental pathway. In the study, the increasing age of adolescents increased the involvement in all the health risk behaviours examined. A finding that is consistent with previous reports is that an increase in age heightens alcohol consumption [45–48], smoking cannabis [45, 46] and sexual activities [48] among high school students. These findings were not unexpected given that increasing age exposes adolescents to life-unfolding adventures that may either build or destroy their life dreams.

In line with previous studies residing in an urban area is associated with an increased risk of using alcohol [35] and smoking cannabis [35, 36]. It contrasts with findings that rural high school children were more likely to consume alcohol [34, 36, 43, 49] and smoke cannabis [34] than their urban peers. Our findings are amenable to the recent development in South Africa. The availability of these substances to urban adolescents’ is a sequel to the fast process of urbanization in South African society. The increased use of alcohol and cannabis by urban adolescents may be due to exposure to these substances which is expected in a society with weak substance use regulations.

Similarly, our findings are in line with some of the previous research that high SES is associated with an increased risk of alcohol consumption [29, 50] and smoking cannabis [51, 52]. Alcohol price is increasing consistently and cannabis is expensive in South Africa. It is logical that greater financial resources among socioeconomically advantaged adolescents’ may explain their use of alcohol and cannabis compared with socioeconomically disadvantaged. However, our findings contradict reports [53] that more socioeconomically disadvantaged states increased smoking among adolescents. Wang et al. [28] on the other hand did not find any association between socioeconomic status and substance use. These discrepancies between the current study and previous studies could be due to different measures used for SES. Previous studies used the educational status [28] and family income [53] of the parents whereas affordability of basic human essential needs was used in the present study.

In this study, negative feelings about the EPEP programme sufficed to increase the risk of alcohol consumption and smoking cannabis. This is counterintuitive to the aim of the school-based programme which was designed to improve learners’ health and self-efficacy attitude to deal with risky environments. The reason for the learners’ displeasure or unable to express their feelings about the programme is not visible from the data. However, there are possible deductions from the assessment of the EPEP programme. Timol et al. [54], suggested that the peer-education programme may have placed too much responsibility on the peer educators. In addition, the use of same-age peer educators was not an effective delivery model. Furthermore, about 20% of peer educators did not meet regularly with the facilitators which could have led to poor programme outcomes and even demoralization. Taking together, school connectedness may be questionable during the EPEP programme implementation. Therefore, further investigation is needed to ascertain the learners’ displeasure to make subsequent school-based peer-education programmes exciting.

Receiving payment from casual work increased the risk of alcohol consumption, smoking cannabis and engaging in sexual intercourse. These findings resonate with those of the National Longitudinal Study on Adolescent Health in the United States which suggests that paid work augments health risk behaviours among adolescents [55]. This suggests that access to finances is a risk to health behaviours among adolescents. It is not known to what extent economic pressure drives these schoolchildren to engage in work for payment. While it is permissible for learners to do casual work with payment, it is also important to educate them on how to invest their earnings in useful ventures. The use of proceeds from causal work by learners to aggravate their involvement in health risk behaviours may suggest a lack of direction and guidance which needs the commitments of the schools and parents. Given that the Department of Education runs school feeding schemes to avert circumstances leading to child labour due to the quest for money, interventions to reduce learners’ engagement in health risk behaviours should consider abolishing casual work with pay.

The findings that participation in school sports increased the risks of alcohol consumption and sexual activities are in agreement with previous studies [56, 57] but in contrast with other reports [55, 58]. Previous studies have shown that school engagements such as sports are protective factors against health risk behaviours [59, 60]. In addition, the expectation of excellent performance in sports with good guidance from educators is anticipated to deter risky behaviours. This may not be the case with the present study. Thus, may imply poor monitoring of the learners in the sports environment and a lack of connectedness with the school environment. The negative relationship between participation in school sports with alcohol consumption and sexual activities is unexpected. Our findings may suggest that sporting activities exposed the learners to negative social environments. This finding implies that learners who participated in school sports are at dual risks of alcohol misuse and sexual negative outcomes.

Cross-cutting all the health risk behaviours studies, we found that participating in religious activities played a central protective role. This protective role of religious participation agrees with other school-based studies on alcohol consumption [48, 50]; cannabis [50] and engaging in sexual activities [48, 50]. Previous studies have demonstrated that irrespective of the dimension of religious measurement, it attenuated health risk behaviours both in small [35, 38] and large studies [48, 50]. Interventionists should consider a religious variable as an important factor in diminishing adolescents’ health risk behaviours.

The findings that being a female is protective against engaging in sexual intercourse agree with previous school-based studies in KwaZulu-Natal [48] and Cape Town [50]. The low likelihood of female learners engaging in sex may suggest a natural reservation towards sensual pleasure-seeking behaviour in a society that frowns at early sexual initiation. It could also be a deliberate action by the female learners to avoid unwanted pregnancies. Taking this together, the present study suggests that males are at risk of contracting sexually transmitted infections due to a higher propensity to engage in sexual intercourse.

The protective effect of the coloured population group on engaging in sexual intercourse seems counterintuitive, but in fact in agreement with other reports [50, 61]. The Association between substance use and increased risk of sexual behaviours is well documented [22, 50]. Contrary to our popular expectation coloured population group exhibited a low risk of engaging in sexual intercourse in the area where the use of the illicit substance is rife. Although we did not assess the association between substance use and sexual activities, the finding appeals to further investigation on the nexus of a population group, substance use and engaging in sex.

Consistent with the previous report [61, 62] family structure of both parents is protective against smoking cannabis. Orientating children to inculcate in them good behaviours often needs the commitment of both parents. Parental monitoring of adolescents has been reported to suppress substance use [63, 64]. Parental monitoring of adolescents’ activities is cumbersome and challenging but can easily be managed when two parents are involved. This finding may constitute family connectedness and good parental guidance.

## Conclusion

The present study is unprecedented in providing the first insights into characterizing risk and protective factors from the peer-education programme intervention among grade 8 learners in the Western Cape. While the EPEP programme was designed to promote positive health behaviour among school learners, the findings of the study have elicited pertinent factors that appeal to consideration in designing school-based intervention programmes within South Africa. For instance, participating in religious activities played a more retarding role in health risk behaviour than the components of the peer-education programme. On the other hand, some background characteristics of the learners were more prominent in promoting health risk behaviour than the peer-education programme. Therefore, the findings will guide the Department of Basic Education and Non-governmental organizations or any other organization that propagates health promotion on predisposing factors that exacerbate or reduce risky health behaviours among adolescents.

### Limitations and strengths of the study

There was 24% sample attrition between the baseline and final data collection. There is the possibility that the respondent would behave differently because of the awareness that they were part of the study. The age of the adolescents was categorized which may result in a loss of precision. However, this is a high-quality longitudinal study that established invariance in the relations between risk and protective factors on adolescent health risk behaviour in South Africa.

## **Recommendation**

The study has characterized protective and non-protective factors on school learners’ disposition to consume alcohol, smoke cannabis and premature engagement in sexual activities. Since the EPEP programme was premised on promoting healthy behaviour among school learners, interventionists should consider making the programme attractive to learners. Furthermore, giving proceeds to learners’ for piece jobs should be discouraged. While learners are encouraged to participate in sports, proper monitoring of learners’ activities in the sporting environment is needed. Since participating in religious activities elicited protection against health risk behaviour, such practices should be encouraged among learners.

## Data Availability

The datasets generated and analysed during the current study are available. The data is available through the HSRC Research Data Service (http://datacuration.hsrc.ac.za/).
